# Assembly and annotation of *Solanum dulcamara* and *Solanum nigrum* plant genomes, two nightshades with contrasting susceptibilities to *Ralstonia solanacearum*

**DOI:** 10.1093/g3journal/jkaf119

**Published:** 2025-05-26

**Authors:** Sara Franco Ortega, Sally James, Lesley Gilbert, Karen Hogg, Harry Stevens, Jason Daff, Ville-Petri Friman, Andrea L Harper

**Affiliations:** Department of Biology, Centre for Novel Agricultural Products, University of York, York YO10 5DD, UK; Department of Biology, University of York, York YO10 5DD, UK; Department of Biology, University of York, York YO10 5DD, UK; Department of Biology, University of York, York YO10 5DD, UK; Department of Biology, University of York, York YO10 5DD, UK; Department of Biology, University of York, York YO10 5DD, UK; Department of Biology, University of York, York YO10 5DD, UK; Department of Biology, University of York, York YO10 5DD, UK; Department of Biology, Centre for Novel Agricultural Products, University of York, York YO10 5DD, UK; Department of Biology, University of York, York YO10 5DD, UK

**Keywords:** bittersweet nightshade, black nightshade, Oxford Nanopore Technologies, methylation frequency, comparative genomics, Orthofinder, genome assembly

## Abstract

To understand why close wild plant relatives of crops, such as *Solanum dulcamara*, are resistant to *Ralstonia solanacearum* we need genome resources to perform comparative studies and identify key genes and pathways. We *de-novo* assembled and annotated the genome of a resistant/tolerant *S. dulcamara* and susceptible *Solanum nigrum* plant using a hybrid approach including Oxford Nanopore Technologies and Illumina sequencing. Comparative genomic analysis was then performed to find differences between the genome of *S. dulcamara* and other susceptible Solanaceous species including potato, tomato, aubergine, and *S. nigrum* and one susceptible and one resistant *S. americanum* accession. We identified genes associated with auxin-transport only in *S. dulcamara* and a collection of pattern recognition receptors (PRRs) in orthogroups only found in resistant/tolerant plant species, which we hypothesize may improve recognition of pathogen-associated molecular patterns associated with *R. solanacearum*. We also identified an arsenal of nucleotide-binding leucine-rich repeat receptors (NLRs) in the *S. nigrum* genome that are shared with the other susceptible species and could be acting as susceptibility factors. Finally, we identified differences in methylation frequency across the gene bodies in both species, which may be associated with epigenetic regulation of resistance. Future work should assess the functional role of these PRRs and NLRs during bacterial wilt development to determine if they could offer potential novel targets for breeding to improve bacterial wilt resistance.

## Introduction

Bacterial wilt disease caused by *Ralstonia solanacearum* is one of the most important plant diseases worldwide, causing important economic and yield losses in more than 400 crops and being especially deadly to the Solanaceae (Mansfield *et al*. 2012). Despite the global efforts to find solutions to this disease, all methods have failed to control this plant pathogen efficiently (Lemaga *et al*. 2001; Nicolopoulou-Stamati *et al*. 2016; Cardenas Gomez *et al*. 2024). Breeding for plant resistance is the most environmentally friendly and sustainable long-term solution and requires identifying quantitative trait loci (QTL) for introgression of genes associated with resistance. QTL analysis has been performed in many crop species affected by this pathogen, including tobacco (Qian *et al*. 2013), peanut (Zhao *et al*. 2016), potato (Habe *et al*. 2019), and chili pepper (Lee *et al*. 2022), as well as tomato utilizing the resistant tomato cultivar Hawaii 7,996 (Wang *et al*. 2013). However, as crop breeding often leads to a loss of genetic diversity, including resistance alleles (Flint-Garcia 2013), wild host plants, which are also infected by the pathogen and frequently used as wild plant reservoirs, may be a valuable source of resistance.

*Solanum dulcamara* L. also known as woody nightshade, climbing nightshade, or bittersweet, is a semi-woody perennial belonging to the genus *Solanum* L., which is formed of more than 1,400 species, including important crops such as tomato, potato, and eggplant. This nightshade is primarily distributed across the Northern Hemisphere and is one of the few *Solanum* species native to Europe. It has a broad global range, thriving in diverse habitats—from riverbanks and lakes to arid environments like dunes. *S. dulcamara* plants exhibit significant phenotypic plasticity, which has complicated their taxonomic classification; currently it belongs to the Dulcamaroid clade (Huang *et al*. 2023), one of the 13-well supported monophyletic clades within the *Solanum* genus. Other nightshades, such as *Solanum nigrum* L. (black nightshade) are commonly found in wooded areas, gardens, vineyards and on river banks (Agriculture and Horticulture Development Board 2025). *Solanum americanum* (American black nightshade) is considered the diploid ancestor of the hexaploid *S. nigrum* (Poczai and Hyvönen 2011), and belongs to the closely-related Morelloid clade. The Potato clade includes important economic crops such as potato (*S. tuberosum*) and tomato (*S. lycopersicum*) (Särkinen *et al*. 2013; Huang *et al*. 2023).

During the winter, bittersweet or black nightshades can act as a natural reservoir of the major plant pathogen *R. solanacearum* (Elphinstone *et al*. 1998; Elphinstone and Matthews-Berry 2020), and they could also be important reservoirs of other plant diseases, for example, *S. nigrum* is known to be a reservoir of tomato yellow leaf curl geminivirus (Bedford *et al*. 1998). Comparativegenomic studies can help to understand the genetic basis of varying levels of susceptibility to bacterial wilt between wild and crop species. However, genomes of wild hosts are often lacking, and hence, obtaining accurate and contiguous genomes is required for comparing and identifying genes linked to disease susceptibility.

The aim of this study was to generate high-quality genome assemblies for a resistant/tolerant *S. dulcamara* accession and a susceptible *S. nigrum* accession using a hybrid approach combining Oxford Nanopore Technologies (ONT) and Illumina sequencing. We sought to produce highly contiguous and complete genomes, with genome size and ploidy confirmed through flow cytometry, and comprehensive de novo genome annotations, which included transposable elements (TEs) and disease-related genes, using mRNA data from various tissues. We then aimed to use these high-quality genome assemblies for comparative genomics, to identify genes uniquely associated with low or high susceptibility to *R. solanacearum.*

In resistant *S. dulcamara*, we identified unique genes with functions related to auxin-transport. We also identified pattern recognition receptors (PRRs), which were only present in the two resistant species used in the analysis, which could be important for pathogen recognition and activation of the plant immune system. Conversely, we identified an arsenal of nucleotide-binding leucine-rich repeat receptors (NLRs) shared between *S. nigrum* and the other susceptible plants species, which could act as susceptibility factors to *R. solanacearum*.

Finally, given the potential roles for DNA methylation in regulating gene expression, encoding stress memory, and modulating defence responses, we aimed to explore DNA methylation patterns by leveraging ONT capacity for methylation quantification. This included assessing methylation across different genomic regions, particularly in genes associated with reduced susceptibility and in relation to TE. Through these objectives, we aimed to deepen our understanding of genetic and epigenetic factors underlying resistance and susceptibility to *R. solanacearum* in these species.

## Material and methods

### Plant growth

*S. dulcamara* and *S. nigrum* seeds were obtained from the Millenium Seed Bank, Royal Botanic Gardens Kew (ID-39084 isolated from the UK in 1982 and ID-170516, isolated from the UK in 2000). The plants were grown and propagated at the University of York (York, UK). Seeds were surface sterilized for 1 min in bleach/water (1:99 v/v), rinsed with distilled water and then stratified for 4 days on wet tissue paper at room temperature. After germination, the seeds were moved to compost (John Innes No2, approximate 125 g compost/pot) in a growth room kept at 20°C (±2°C) and with 14 h/10 h light/dark conditions with the lights (Model L28, Valoya, Loughborough, UK) suspended approximately 40 cm above the crop, which provide a light intensity of about 120 µmol m^−2^s^−1^ (SD ±10). The plants were self-pollinated to obtain berries.

### Plant inoculations with *R. solanacearum*

To assess the susceptibility of both *S. dulcamara* and *S. nigrum* to *R. solanacearum*, plant inoculations were performed and compared with a susceptible tomato cultivar “Moneymaker” (Franco Ortega *et al*. 2024). The three plant species were grown as above for 17 days. At 17 days (4 days before the inoculation), plants were moved to a PHCbi growth cabinet 24°C 16 h light, 20°C 8 h dark, with light intensity reaching 220 µmol m^−2^s^−1^ measured from the center of the cabinet (3 Panasonic FL40SSENW37 fluorescent tubes (37W/5700K)/side on 3 sides of the cabinet) to acclimate. Bacterial inoculum was prepared by growing *R. solanacearum* Race 3 Biovar 2 strain UW551 stock in CPG liquid media (Casamino acid 1 g/L, Peptone 10 g/L, Glucose 15 g/L) at 28°C for 3 days, maintaining agitation at 100 rpm. 24 h before plant infections, fresh CPG sterile media (10% of the volume of the inoculum) was added to the bacterial cultures. The OD_600_ was adjusted to 0.7 (8.3E + 09 cells) before 5 mL of the bacterial inoculum was added as a root drench (21 day-old plants). A total of 18 plants/species were inoculated with the pathogen, and the same number of plants/species were maintained as negative controls by adding 5 mL of sterile CPG media onto roots. The symptoms were recorded for 21 days post-infection using a scale of 0–4, where 0 = plants with no wilting symptoms, 1 = 25% of the plant surface showing symptoms, 2 = 50% of the plant showing wilting symptoms, 3 = wilting symptoms in 75% of the plant and 4 = dead plants. Curves of bacterial wilt disease progression and bar plots reporting the number of healthy (DI = 0) or diseased plants (DI > 0) for each plant species were plotted using ggplot2 package in R (Wickham 2016).

### Plant material for genome assembly

DNA was extracted from 3-month-old adult plant leaves using the high molecular weight gDNA extraction using the protocol described in Vaillancourt and Buell (2019), which uses Carlson buffer and the QIAGEN Genomic-tips 500/G (QIAGEN, Manchester, UK). The gDNA quality was assessed with a Nanodrop and Agilent Tapestation 4,200 (Agilent, Cheadle, UK). The *S. dulcamara* gDNA library was prepared using ONT ligation library preparation kit LSK109 and run using R9.4.1 flowcells on a PromethION 24 by the Bioscience Technology Facility, York. The same gDNA was also sequenced using the NovaSeq 6,000 platform (Illumina, CA, USA) with a paired-end library strategy (PE150) provided by Novogene UK (Cambridge, UK). The *S. nigrum* genome was obtained using a ONT's ligation kit LSK114 and run on PromethION R10.4.1 flowcells, keeping only the high-accuracy reads following superaccuracy basecalling with guppy software version 6.4.6.

For *de-novo* annotation, we collected roots, stems, leaves, flowers and berries from each plant at the same time as the DNA samples, and these were stored at −70°C prior to RNA extraction. The plant material was ground in liquid nitrogen and the RNA extracted using the E.Z.N.A. Plant RNA Kit (VWR, Lutterworth, UK), which included DNase treatment to remove residual DNA according to the manufacturer's instruction. The quality of the RNA was assessed with Nanodrop, Qubit 4.0. (Thermo Fisher Scientific, Milton Park, UK) using the Qubit RNA Broad Range kit (Thermo Fisher Scientific) and the Agilent Technology 2,100 Bioanalyzer (Agilent Technologies, CA, USA). Only samples with RNA integrity number >6 were used to prepare the cDNA libraries. For the *S. dulcamara* genome annotation, full length barcoded cDNA libraries were generated for each tissue using ONT's cDNA barcoding kit PCB109, and barcoded cDNA libraries were pooled for running on a R9.4.1 flowcell on a PromethION 24. The same RNA was used to for short read sequencing by Novogene services (Cambridge, UK) using the NovaSeq 6,000 platform, a paired-end strategy (PE150) and an Illumina mRNA library preparation (poly A enrichment). For the *S. nigrum* genome annotation, we obtained RNA from the same tissues collected at the same time points as with *S. dulcamara* but sequenced them only with the Illumina NovaSeq 6,000 platform, using equimolar concentrations of the 5 pooled tissues.

### Chloroplast and mitochondrial genome assembly

The *S. dulcamara* chloroplast assembly was started by using minimap2 to map the raw ONT reads against the complete *Solanum lycopersicum* chloroplast (NC_007898.3), followed by *de-novo* assembling the mapped reads using CANU (version 2.1.1) (Koren *et al*. 2017) with setting genomeSize = 155k. RACON version 1.4.20 and MEDAKA version 1.0.3 were further used to polish the first preliminary chloroplast assembly (Vaser *et al*. 2017). To improve the assembly, the same ONT reads were mapped again against this preliminary chloroplast assembly followed by a second round of assembly using CANU, RACON, and MEDAKA. Unmapped reads obtained after the second mapping round for the chloroplast, were then mapped using minimap2 against the *Solanum lycopersicum* mitochondrion complete genome (NC_035963.1), and mapped reads were used to assemble the first preliminary *S. dulcamara* mitochondrial genome following the same approach with CANU, RACON, and MEDAKA. As with the chloroplast assembly, this preliminary mitochondrion assembly was followed by a second round of assembly as before.

The *S. nigrum* chloroplast and mitochondria were directly assembled using minimap2 by mapping the raw ONT reads to the *Solanum lycopersicum* chloroplast (NC_007898.3) and *Solanum lycopersicum* mitochondrion complete genome (NC_035963.1), with one round and two rounds of assembly with CANU, RACON, and MEDAKA, respectively.

### Flow cytometry for determining genome size and ploidy with *S. dulcamara* and *S. nigrum*

To assess the nuclear genome size and ploidy of both *S. dulcamara* and *S. nigrum*, we used a flow cytometry CytoFlex LX (Beckman Coulter) and followed the protocol by Doležel *et al* (2007) using TrisMgCl_2_ buffer for the homogenization step. *Solanum lycopersicum* (tomato), and *Zea mays* (maize) were used as references to estimate the genome sizes. The protocol was performed with a total of 20 mg of leaves using 10 mg of the target and 10 mg of the reference. CytExpert software was used to analyse the results.

### Nuclear genome assemblies

Different approaches were used to assemble the nuclear genomes for each plant species to obtain the most contiguous and complete genome assemblies. After assembly of the chloroplast and mitochondrial genomes, CANU (version 2.1.1; Koren *et al*. 2017) was used to assemble the nuclear genome using the unmapped reads. To phase the genome, PURGE HAPLOTIGS (Roach *et al*. 2018) reassigned contigs to haplotigs according to read depth and homology. Finally, the *S. dulcamara* genome was polished with Illumina data using PILON version 1.24 (Walker *et al*. 2014). The *S. nigrum* genome was assembled with FLYE (Kolmogorov *et al*. 2019) using the high-accuracy reads from the PromethION R10.4. run, followed by RACON version 1.4.20 and MEDAKA version 1.0.3. (Vaser *et al*. 2017) to polish the genome using the same high-accuracy reads. Lastly, PURGE HAPLOTIGS was used to reassign contigs to haplotigs.

SEQKIT v2.3.1 was used to assess the assembly size and N50. Completeness of the genome was assessed with the Benchmarking Universal Single-Copy Orthologs (BUSCO version 5.5; Manni *et al*. 2021) against the Solanales and Viridiplantae databases.

Pseudochromosomes (2*n* = 24 for both plant species) were also reconstructed using a similar approach as in Franco Ortega *et al*. (2025) using NTJOIN (version 4.2.1; Coombe *et al.* 2020) with *k* = 16 and *w* = 500 for the *S. dulcamara* and *k* = 32, *w* = 200, and *n* = 2 options for the *S. nigrum* using the previously assembled *S. dulcamara* genome (Christenhusz 2023) as reference. Chord plots were obtained by first mapping with NUCMER 4.0.0 (Kurtz *et al.* 2004) using the options -c 500 -b 100 -l 1000 between the two *S. dulcamara* genomes or -c 32 -b 100 -l 100 between the *S. dulcamara* used as references and the *S. nigrum* pseudochromosomes followed by filtering to maintain only synteny regions with high identity ≥93% and with a minimum length of 1000 bp. The plots were obtained using the circlize package in R.

To annotate the genomes, repetitive elements were identified using REPEATMODELER (version 2.1) and REPEATMASKER (version 2.1; Flynn *et al*. 2020). BRAKER (version 1.9) (Brůna *et al*. 2021) was used to predict the gene models in the masked genome. Protein data from the Viridiplantae (downloaded November 2021; https://v100.orthodb.org/download/odb10_plants_fasta.tar.gz; Zdobnov *et al*. 2021) was used in combination with ONT and Illumina mRNA-Seq data for *S. dulcamara,* and only Illumina for *S. nigrum*. TSEBRA (Gabriel *et al*. 2021) with keep_ab_initio configuration was used to combine the results of both proteins and mRNA-Seq predictions. Annotations were filtered by structure and function using GFACS (Version 1.1.2); Caballero and Wegrzyn (2019) and ENTAP (Hart *et al*. 2020). EGGNOG (Huerta-Cepas *et al*. 2019; Cantalapiedra *et al*. 2021) was used for the functional characterization of the gene models. NLRs genes were annotated with NLR-Annotator v2.1 (Steuernagel *et al*. 2020) using the newly assembly genomes and then using Bedtools v.2.30.0 intersect function to match with the new annotation of each plant species.

### Identification of methylation patterns and TE in plant genomes

To identify potential epigenetic regulation of defence genes in these wild species, we assessed DNA methylation and methylation frequency of the *S. dulcamara* and *S. nigrum* genomes using f5c v1.2 (Gamaarachchi *et al*. 2020) setting the “pore” option to r9 or r10. The frequency was only calculated when the log_lik_ratio (log-like methylated—log-like unmethylated) had a positive value, supporting methylation. TE and tandem repeats were identified using the Extensive *de-novo* TE Annotator (EDTA; Ou *et al*. 2019). For each plant species, the methylation frequency was assessed across three genomic locales: the gene body, 1 kbp upstream and 1 kbp downstream considering all the genes in the genomes. TEs were classified into terminal inverted repeat sequences (TIRs), non-TIRs or long terminal repeats (LTRs). Plots representing average methylation frequency across the genomic locales in function of the TE type (or genomic locales without TEs), were obtained by dividing the gene body length into 20 intervals each representing 5% of the gene's length, whilst the upstream and downstream regions were divided into 20 intervals corresponding to 50 bp each. Methylation frequency was also assessed for specific genes identified during the comparative genomics analysis. To assess if the methylation frequency was conserved between these two plant species, we performed Pearson correlations between the methylation frequency of *S. dulcamara* and *S. nigrum* in the three genomic locales and plotted them with ggscatter from the R package ggpubr (Kassambara 2023).

### Comparative genomics to find orthogroups and shared genes between plant species with contrasting susceptibility to *R. solanacearum*

To find genes present only in plant species with lower susceptibility to bacterial wilt, we used the nightshade genomes assembled here, along with the genomes of *S. tuberosum* C88 (http://spuddb.uga.edu/data/), tomato (pangenome of *S. lycopersicum*; Zhou *et al*. 2022), aubergine *S. melongena* (https://solgenomics.net/ftp/genomes/Solanum_melongena_V4.1), and two accessions of *S. Americanum:* SP2773 and SP2775 (Moon *et al*. 2021). The 7 genomes were inputted into the Synteny Imaging tool (SYNIMA; Farrer 2017) using the Orthofinder pipeline (Emms and Kelly 2019). The unrooted species tree was produced using the STAG (Species Tree inference from All Genes) algorithm (Emms *et al*. 2018) using the command Orthologs_to_summary.pl. After obtaining the Orthogroups.csv file, we used kinfin (version 3; Laetsch and Blaxter 2017) to calculate the number of orthogroups shared between the plant species. The R package UpSetR (Conway *et al.* 2017) was used to plot the orthogroups shared between species. We also retrieved genes belonging to orthogroups only present in *S. dulcamara,* and those also shared with the resistant *S. americanum* SP2773 accession and those in common between the rest of the susceptible species (*S. nigrum*, *S. americanum* SP2775, *S.lycopersicum*, *S. melongena*, and *S. tuberosum*). To understand the function of the genes only present in species with low susceptibility to bacterial wilt, the *S. dulcamara* genes in these orthogroups were retrieved and a gene ontology (GO) enrichment analysis was preformed using the topGO package (Alexa and Rahnenfuhrer 2024) in R, using the whole genome annotation of *S. dulcamara* as the universe. We further utilized the annotations to identify resistance-associated NLRs by focusing on those found exclusively in *S. dulcamara*-specific orthogroups or shared with *S. americanum* SP2773. In addition, we identified potential susceptibility factors as NLRs in orthogroups found exclusively in *S. nigrum* or shared with other susceptible plant species. Methylation of PRRs was assessed as explained above, whilst the methylation plots of NLRs shared between *S. nigrum* and susceptible plant species were obtained by representing average methylation frequency across the genomic locales, dividing the gene body length into 6 intervals each representing 15% of the gene's length, whilst the upstream and downstream regions were divided into 6 intervals corresponding to 166 bp each.

### Gene expression of selected PRRs

The mRNA-Seq used for the *S. dulcamara* annotation was also used to evaluate the expression of selected PRRs (Sd_g3085.1, Sd_g3087.1, Sd_g3090, Sd_g26139.3, Sd_g8870, Sd_g28574), identified during the comparative genomic analysis with other plant species. Salmon v.1.10 (Patro *et al*. 2017), was used to obtain transcript-per-million (TPMs) in each of the 5 plant tissues (roots, stems, leaves, flowers, and berries) using the long ONT reads and default parameters. The expression of these genes was also compared with other PRRs (leucine-rich repeat receptor-like kinases; LRRs). Plots of log (TPMs + 1) were obtained using ggplot2 in R.

## Results and discussion

### Comparing plant susceptibility to *R. solanacearum*

**Fig. 1.**
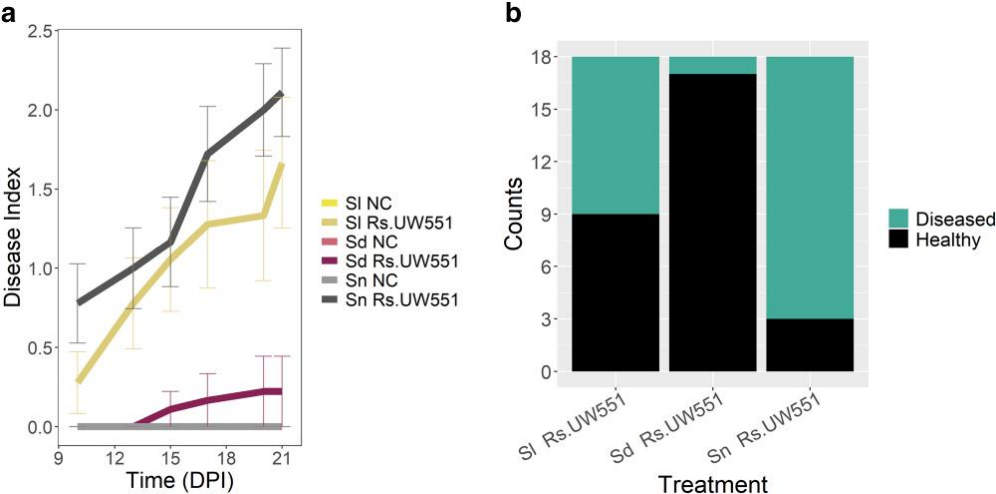
Disease index at 21 days post-inoculation of *S. dulcamara*, *S.nigrum*, and tomato plants confirming the resistance/tolerance mechanisms of nightshades against *R. solanacearum* at 24°C. a) Disease index at 21 dpi of *S. lycopersicum* negative control plants (*N* = 18) in light yellow (Sl NC); *S. lycopersicum* inoculated with *R. solanacearum* strain UW551 (*N* = 18) in dark yellow (Sl Rs.UW551); *S. dulcamara* negative control plants (*N* = 18) in pink (Sd NC); *S.dulcamara* inoculated with *R. solanacearum* strain UW551 (*N* = 18) in dark red (Sd Rs.UW551); *S.nigrum* negative control plants (*N* = 18) in light gray (Sn NC); *S. nigrum* inoculated with *R. solanacearum* strain UW551 (*N* = 18) in dark gray (Sn Rs.UW551). Error bars are represented for each time point for each plant species when the disease scores where measured. b) Number of diseased and healthy plants in each treatment (18 plants inoculated/species) representing *S. lycopersicum*, *S. dulcamara*, and *S. nigrum* inoculated with *R. solanacearum* strain UW551 (Sl Rs UW551; Sd Rs.UW551; Sn Rs.UW551).

We first compared the susceptibility of *S. dulcamara*, *S. nigrum* and tomato to *R. solanacearum* infections. Only 1 out of 18 *S. dulcamara* plants showed bacterial wilt symptoms at 21 dpi, compared with 9 plants showing wilting symptoms in tomato cv Moneymaker, 3 of them with disease index 4 and the rest with disease index 3 at 21 dpi (Fig. 1a and b). In contrast, at 21 dpi, *S. nigrum* showed high susceptibility to *R. solanacearum* with 15 out of 18 plants showing bacterial wilt symptoms, including one dead plant (DI = 4) and 7 plants with a disease index of 3 (Fig. 1b). Overall, this experiment confirmed that these *S. nigrum* and *S. dulcamara* accessions have contrasting levels of susceptibility to *R. solanacearum* at 24°C. *S. dulcamara* showed almost no symptoms after three weeks post-infection confirming tolerance or partial resistance, as reported previously by Sebastià *et al*. (2021).

### Genome assembly and annotation

To identify genes or pathways associated with different susceptibility to *R. solanacearum*, we started by *de-novo* assembling the genome of the partially resistant/tolerant *S. dulcamara* and susceptible *S. nigrum*. We started by assessing genome size with flow cytometry, followed by hybrid assembly of the genome using ONT and Illumina data.

Flow cytometry estimated the *S. dulcamara* genome to be 1.03 Gbp for *S. dulcamara.* Recently Christenhusz (2023) reported a *S. dulcamara* genome size of 1.21 Gbp by flow cytometry.

The *S. dulcamara* chloroplast genome was assembled into a single contig with 226,924 bases (>200× coverage considering a size of 155 kbp), while the mitochondrial genome was assembled using 2,785 reads (91,027,879 bases; >200 times coverage) into 18 contigs covering a total length of 457,235 bp (N50 = 54,728 bp).

After mitochondrial genome assembly, the remaining 1,048,676 reads (26,787,058,484 bases, N50 = 34,429) provided 26× coverage of the *S. dulcamara* nuclear genome, based on the genome size estimated using flow cytometry. After phasing and polishing with Illumina data, the genome assembly constituted a total of 3,356 contigs covering 881,223,639 bp (N50 = 421,481 bp, Supplementary Table 1). BUSCO scores reached 89.9% for Solanales and 94.4% for Viridiplantae databases (Table 1). We then used the *S. dulcamara* genome reported by Christenhusz (2023), with a total length of 946.3 Mb in 105 sequence scaffolds, as a reference to assembled 3,169 contigs out of the 3,356 of the *S. dulcamara* genome reported here into 12 pseudochromosomes (total length of 992,072,612 bp; N50 = 84,035,567; number of undetermined bases = 124,802,331, Supplementary Table 2), consistent with the base chromosome number for *Solanum spp*., and confirming high synteny between the two *S. dulcamara* genome assemblies (Supplementary Fig. 1a).

**Table 1.** BUSCO scores of the new *S. dulcamara* and *S. nigrum* genomes.

	*S. dulcamara*				*S. nigrum*			
	Viridiplantae		Solales		Viridiplantae		Solales	
	Number	Percentage	Number	Percentage	Number	Percentage	Number	Percentage
**Complete BUSCOs (C)**	401	94.40%	5349	89.90%	423	99.60%	5691	95.70%
**Complete and single-copy BUSCOs (S)**	391	92.00%	5394	86.40%	407	95.80%	5513	92.70%
**Complete and duplicated BUSCOs (D)**	10	2.40%	208	3.50%	16	3.80%	178	3.00%
**Fragmented BUSCOs (F)**	8	1.90%	65	1.10%	1	0.20%	55	0.90%
**Missing BUSCOs (M)**	16	3.70%	536	9.00%	1	0.20%	204	3.40%
**Total BUSCO groups searched**	425		5950		425		5950	

On the other hand, flow cytometry reported a1.29 Gbp genome size for *S. nigrum*, suggesting that this *S. nigrum* accession was diploid. The *S. nigrum* genome was then assembled using ONT data (83,200,377,815 bp; N50 = 19,765, 69× coverage), obtaining 3,010 contigs, totaling 1,061,442,536 bp (N50 = 1,883,648, Supplementary Table 1). BUSCO scores reached 95.7% for Solanales and 99.6% for Viridiplantae databases (Table 1). Using the same *S. dulcamara* genome by Christenhusz (2023) as a reference genome, 654 out of 3,010 contigs were assembled into 12 pseudochromosomes (total length of 1,305,681,424 bp; N50 = 111,432,655; number of undetermined bases = 448,566,684, Supplementary Table 2). The low number of contigs and low synteny between both genomes (Supplementary Fig. 1b) suggested major differences between *S. dulcamara* and *S. nigrum* genomes. The *S. nigrum* chloroplast genome was assembled into 7 contigs (N50 = 190,205) with 2,094 reads (31,004,936 bases; ∼200× coverage) consisting of 263,207 bases. The mitochondrial genome was assembled into 4 contigs (N50 = 102,821) using 5,081 reads (90,008,286 bases; ∼200× coverage considering a size of 450 kbp), and resulting in a final length of 396,930 bp.

The GC content average was 35.65% for *S. dulcamara* and 36.54% for *S. nigrum.* De-novo annotation resulted in a total of 27,429 genes (23,800 with functional annotation), which expanded on the *S. dulcamara* de novo transcriptome of 24,193 unigenes reported by (D’Agostino *et al*. 2013). On the other hand, the number of annotated genes for *S. nigrum* reached 39,466 (30,426 with functional annotation), which was fewer than the transcriptome of 47,470 unigenes reported by (Heo *et al*. 2022) using an *S. nigrum* accession obtained from NIBR, Incheon, Republic of Korea. This variation likely reflects substantial diversity among isolates or cultivars from different geographical origins, highlighting the need for pangenome approaches to capture comprehensive gene repertoires in wild species. Notably, we identified 239 and 434 genes classified as NLRs in S. *dulcamara* and *S. nigrum* genomes, respectively.

### *S. nigrum* showed higher DNA methylation frequency across the genome

**Fig. 2.**
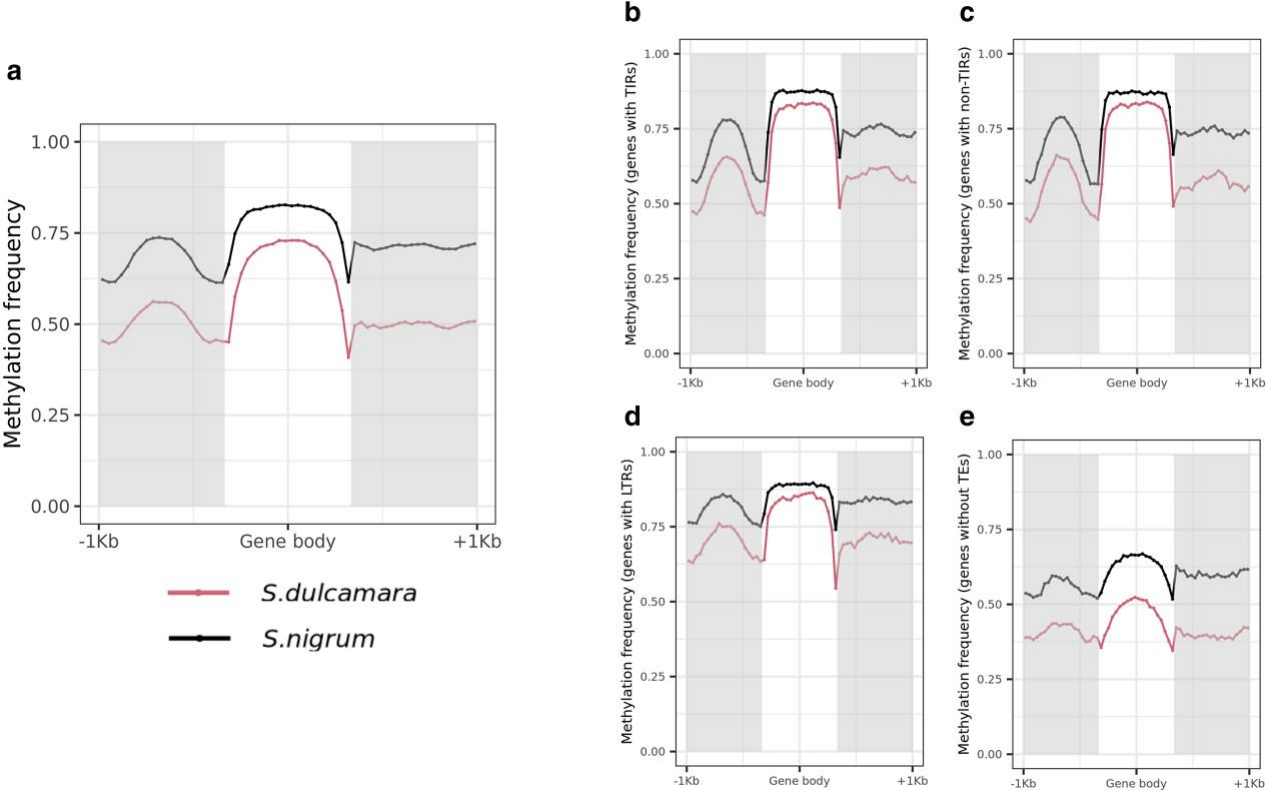
Methylation and TEs across gene body and upstream and downstream regions considering all the genes in the genomes. a) Methylation frequency across gene body, 1 kbpp upstream and 1 kbpp downstream for all the *S. dulcamara* (pink) and *S. nigrum* (black) genes. b—d). Methylation frequency across gene body, 1 kbpp upstream and 1 kbpp downstream for all the *S. dulcamara* (pink) and *S. nigrum* (black) genes containing TIRs b), non-TIRs c) and LTRs d) and genes without TEs e).

Epigenetic changes such as DNA methylation are regulatory mechanisms that play a key role in plant-pathogen interactions, for example by modulating gene expression of defense-related genes (Wu and Fan 2025). We started by assessing whole genome methylation frequencies in both plants and associating these frequencies with the type and content of TEs as they can be associated with silencing (due to hypermethylation) of the genes they are located inside or next to (Lee *et al*. 2023). The methylation frequency in the *S. dulcamara* contigs was 77.4% with an average of 23.4 called sites per contig and 18.11 methylated called sites (Supplementary Table 3). The methylation average increased to 87.5% methylation frequency on the *S. nigrum* genome (with an average of 15.9 methylated sites out of an average of 34.9 called sites; Supplementary Table 4). When the methylation frequency was assessed across different gene locales, it followed similar patterns for both plant species, but it was 17.1% higher in *S. nigrum* than in *S. dulcamara* in the 1 kbp upstream region (67.3% vs 50.2% average), 13.7% in the gene body (78.9% vs 65.9%), and 21.5% 1 kbp downstream region (71.3% vs 49.9%) (Fig. 2a). Using different ONT flow cells might explain these differences, however, Ni *et al*. (2023) recently confirmed the average proportion of 5mC called using R9.4.1 and R.10.4 flow cells is similar (67.44 and 67.70%, respectively), with strong Pearson correlation (0.64) between the results of both in various gene locales. However, the authors confirmed that R10.4 showed a lower background than R.9.4.1 in regions with high GC content Ni *et al*. (2023).

Considering the Ni *et al*. (2023) results, we concluded the differences in methylation frequency between both genomes can only be partially explained by using different flow cells for sequencing. Instead, the overall higher methylation frequency in the *S. nigrum* genome could be associated with different epigenetic regulation, for example, modulation by TEs, which are usually repressed by epigenetic silencing via hypermethylation. TEs also constitute a large part of plant genomes and are essential for the adaptative evolution of a species (Wyler *et al*. 2020; Chu *et al*. 2024).

We confirmed that the percentage of TEs was lower in *S. dulcamara* (948,592 TEs masking 62.86% of the genome) than in *S. nigrum* (881,028 TEs masking 72.94% of the genome). When looking at the distribution of TEs in different genomic locales (gene body, 1 kbp upstream and 1 kbp downstream areas considering all genes in the genome), we observed that *S. nigrum* had more TEs than *S. dulcamara* in all locales. On average across the 27,429 genes in *S. dulcamara*, we observed 1.18 ± 1.24, 2.19 ± 4.26, and 0.96 ± 1.16 TEs/gene for upstream, gene body, and downstream regions, respectively. These numbers increased to 1.67 ± 1.50, 2.87 ± 5.12, and 1.4 ± 1.41 TEs/gene for upstream, gene body and downstream regions, respectively in the *S. nigrum* genome (39,446 genes). As TEs are often hypermethylated, greater numbers of TEs in the *S. nigrum* genome may explain the higher methylation frequency observed in comparison to *S. dulcamara*.

We observed higher methylation frequencies in *S. nigrum* for all TE types in gene bodies (78.2% in *S. dulcamara* vs 85.1% in *S. nigrum* for gene bodies with TIRs; 78.4% vs 85% for gene bodies with non-TIRs and 80.9% vs 87.3% for gene bodies with LTRs; Fig. 2b–d), suggesting that not only are there more TEs in the *S. nigrum* genome, but they are generally more highly methylated in this species. Similar patterns were observed in upstream regions (55.7% vs 67.6% for upstream regions with TIRs; 54.8% vs 67.5% for upstream regions with non-TIRs and 69.2% vs 80.6% for upstream regions with LTRs), and downstream regions (59.5% vs 74.2% for downstream regions with TIRs; 56.9% vs 73.8 downstream regions with non-TIRs and 69.2% vs 83.5% for downstream regions with LTRs; Fig. 2b–d). However, in genes without TEs, methylation frequency was reduced to 46.1 and 62.6% in gene bodies, 40.7 and 55.4% in the upstream region and 39.9 and 60.2% in the downstream region for *S. dulcamara* and *S. nigrum*, respectively. This indicates that the increase in methylation observed in *S. nigrum* is a general feature which is not restricted to TEs.

The methylation frequency of all genes was positively correlated in these two plants (Supplementary Fig. 2), meaning they followed similar methylation frequency patterns despite the higher percentage methylation observed in the *S. nigrum* genome. All Pearson correlations were significant, but they were stronger for the upstream and gene bodies locales for the three TE types than the downstream regions or in genes without TEs (Supplementary Fig. 1), suggesting both upstream and gene bodies showed conserved methylation patterns. These results confirmed that the higher methylation observed in *S. nigrum* (Fig. 2) was likely not due to different ONT flow cells used for sequencing but could be partially associated with both a greater accumulation of TEs in *S. nigrum*, and increased methylation of TEs within gene regions.

Overall, we obtained two new contiguous genomes and showed differences in methylation frequencies across the genomic locales that might be linked with higher TE percentage in *S. nigrum* than in *S. dulcamara*. Differences in epigenetics might have adaptative and evolutionary consequences that should be explored in future work.

### Comparative genomics confirms the presence of genes related to immunity and phytohormones only in resistant/tolerant accessions

To identify genes associated with bacterial wilt resistance/tolerance, we performed a comparative genomics analysis with 7 plant species (resistant/tolerant species: *S. dulcamara* and *S. americanum* SP2773 and susceptible species: *S. nigrum*, *S. americanum* SP2775, *S. lycopersicum*, *S. melongena*, and *S. tuberosum*) focusing on the Orthofinder results. A total of 449,192 genes were evaluated, with 398,266 genes assigned to 29,498 orthogroups and 50,926 genes not assigned to orthogroups.

A high percentage of the genes (>83%) (Supplementary Tables 5–7) in each species were assigned to an orthogroup, while unassigned genes (those that did not produce a good BLAST hit against the full transcriptome dataset using the default SYNIMA Blast_grid_all_vs_all.pl script parameters) were relatively few (<17%), indicating that most genes had been retained in at least some of the species. In total, we obtained 12,574 (42.6%) orthogroups with all species present, 5,549 species-specific orthogroups (which contained multiple paralogs in one species), and 51 single-copy orthogroups (i.e. containing exactly one gene copy in each species) (Supplementary Table 6). The median size was 10 genes per orthogroup.

**Fig. 3.**
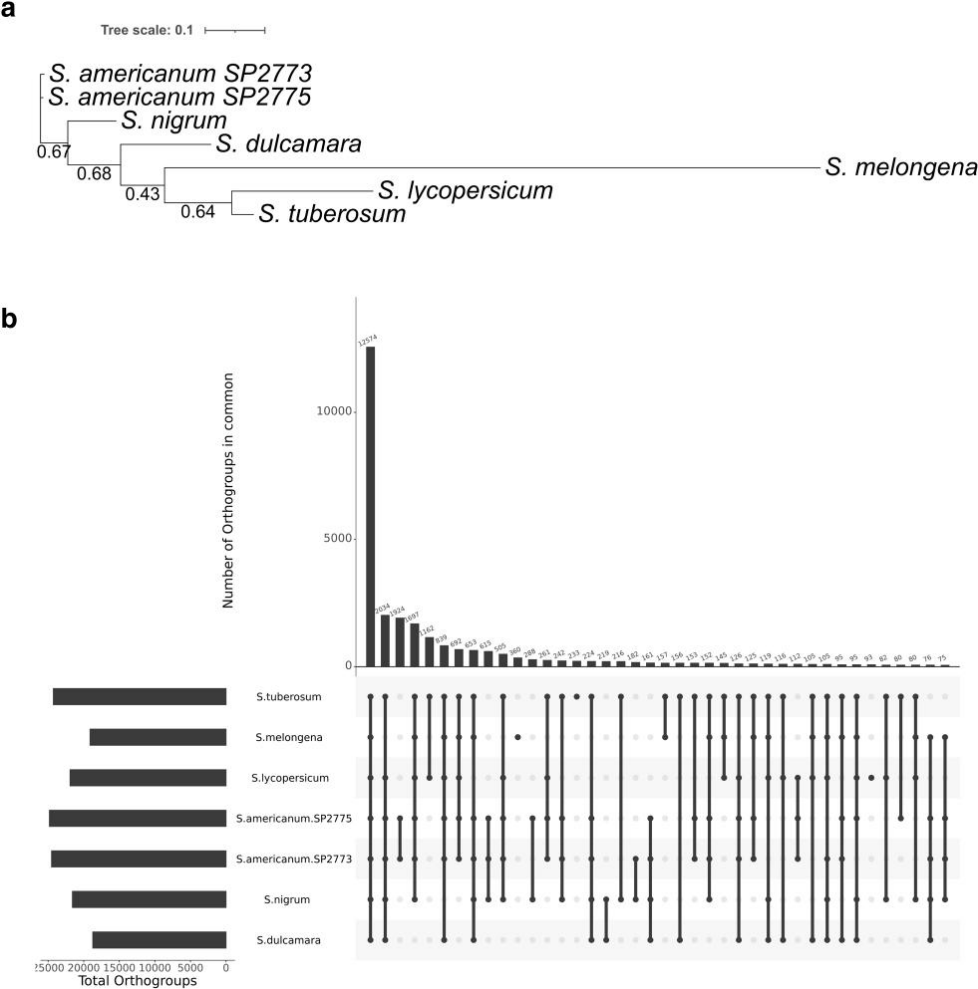
Species tree and orthogroups shared between *S. dulcamara* and *S. nigrum* and other susceptible and resistant species to *R. solanacearum*. a) Unrooted species tree was produced using the STAG (Species Tree inference from All Genes) algorithm between plant species and accessions with different susceptibility to *R. solanacearum* (resistant/tolerant species: *S. dulcamara* and *S. americanum* SP2773 and susceptible species: *S. n*i*grum*, *S. americanum* SP2775, *S. lycopersicum*, *S. melongena*, and *S. tuberosum*) created by Orthofinder and assessing the CDS models of each species. b) Orthogroups intersect between the plant species and accessiones used in A assessed with Orthofinder. Only groupings with 75 orthogroups are represented in the plot.

A high number of orthogroups (219) were shared between *S. nigrum* and *S. dulcamara* only (Supplementary Table 7, Fig. 3), while 27 orthogroups were only present in *S. dulcamara* (0.3% or 90 genes), and *S. nigrum* had 61 unique orthogroups (1.2% or 471 genes; Supplementary Table 8) while the susceptible species (*S. nigrum*, *S. americanum* SP2775, *S. lycopersicum*, *S. melongena*, and *S. tuberosum*) shared 3,531 orthogroups. The unrooted tree produced by orthoFinder (Fig. 3) grouped the *S. americanum* samples closely together and placed all Potato clade commercial crops (*S. tuberosum*, *S. lycopersicum*, and *S. melongena*) into a clade nested within a larger clade containing *S. nigrum* and *S. dulcamara*, supporting previous classifications (Huang *et al*. 2023) and the accuracy of both genome assemblies. Susceptible and resistant/tolerant plant species/cultivars were dispersed across the tree and did not group together.

To identify potential mechanisms underpinning the ability of *S. dulcamara* to resist *R. solanacearum*, we first explored 90 genes belonging to 27 orthogroups that were only present in *S. dulcamara* (Supplementary Table 9) using singular enrichment analysis of GO terms. This revealed an enrichment of auxin transport-related GOs (Supplementary Table 10). In other bacterial plant disease systems, salicylic acid defence responses stabilize auxin repressors, translating into boosted disease resistance when auxin responses are blocked (Wang *et al*. 2007), and during *R. solanacearum* infections, resistant tomato plants repress the transport and signaling of auxins (French *et al.* 2018). Auxin accumulation, depending on transport of PINs (Grieneisen *et al*. 2007), also explains *R. solanacearum*-related alteration of root development in the susceptible plant *Arabidopsis thaliana*, which facilitates the colonization of the plant (Zhang *et al*. 2024). The enrichment of genes associated with auxin transport in the partially resistant *S. dulcamara* may have a potential role in facilitating pathogen colonization and establishing latent infections (Sebastià *et al*. 2021). However, it is also possible that these genes are involved in boosting root development and could be associated with resistance, as increases in root growth and area have been seen in resistant crops when inoculated with *R. solanacearum* (Meline *et al*. 2022). However, this hypothesis should be further explored with this wild plant in the future.

We also assessed the 30 genes found in common between the two resistant accessions: *S. dulcamara* and *S. americanum* SP2773 (Supplementary Table 11). Here we observed: Sd_g8870, a LRR orthologous to *Arabidopsis thaliana* At1g35710; and Sd_g28574, a LRR receptor-like serine threonine-protein kinase orthologous to At1g07650, which has previously been found to be differentially expressed in susceptible potatoes when inoculated with *Dickeya solani*, the causal agent of blackleg and soft rot in potato (Hadizadeh *et al*. 2022). These genes are often categorized as PRRs which recognize pathogen-associated molecular patterns (PAMPs) inducing PAMP-triggered immunity in infected plants (Yuan *et al*. 2021). These genes could be used as targets in future works to understand their functional role during bacterial wilt disease development and confirm if they are associated with resistance/tolerance.

Overall, the comparative genomic analysis revealed a specialized group of PRRs in species that are resistant/tolerant to *R. solanacearum* and not present in other susceptible crops and wild plants. These PRRs could enable these plants to recognize and defend against a wider range of pathogens. We also identified increased numbers of auxin-transport related genes in *S. dulcamara*, which may be linked to their role as a reservoir host to this pathogen or in boosting resistance.

### Genes common to resistant/tolerant plant species show different patterns of methylation frequency and TEs

After identifying PRRs only present in *S. dulcamara* and in the resistant *S. americanum* SP2773, we assessed if these potential resistance-associated genes showed higher numbers of TEs or different DNA methylation frequencies across the three genomic locales (upstream, gene body, and downstream regions). As we did not have the methylation profile of *S. americanum* SP2773, we only assessed methylation frequencies and TE content in *S. dulcamara*.

We observed that Sd_g8870 and Sd_g28574 showed higher methylation in the upstream and downstream regions than other LRRs (Supplementary Table 12). Sd_g8870 showed 47.6% methylation frequency in the upstream region (compared with 39.4 ± 29.4 in other LRRs; *P* = 0.013), while Sd_g28574 showed 56.9%, which is significantly higher than the average seen in other LRRs (*P*-value = 9.6E−07). Similarly, in the downstream region of the gene, Sd_g8870 showed higher methylation than the average in other LRRs (the average in other LRRs is 47.8 ± 28.9; *P*-value = 1.36E−10). Instead, the methylation frequency in the gene body was lower 27.3% for Sd_g28574, *P*-value = 2E−10; 38.1% for Sd_g8870, *P*-value = 0.002 in both cases than the average (48.9 ± 29.3).

To confirm if these methylation frequencies were associated with transcriptional changes, we evaluated the expression of these specific genes and compared them with the expression of other LRRs. We hypothesized that higher methylation frequency in the promoter region would be associated with lower expression, and lower methylation frequency in these upstream regions would be associated with higher expression. In this case, the expression of these two genes was zero in roots (Supplementary Fig. 3), which could be due to the higher methylation observed in upstream regions. We also observed that Sd_g8870 had a higher number of TEs than average in the upstream region (genome average is 0.24 ± 0.60, *z* = 208.56, *P* < 0.001; Supplementary Table 12), which could be affecting methylation frequency and consequently the gene expression. However, as we measured the methylation frequency using leaves, future experiments should look to see if the methylation profiles of other tissues, such as roots, correlate with the methylation frequency measured in leaves.

Overall, these results could indicate the expression of redundant disease-related genes, especially in roots, being the point of entry of *R. solanacearum,* is being suppressed by DNA methylation in upstream regions. These results are in line with the results seen by Kong *et al*. (2018) who suggested other disease resistance (*R*) genes such as NLR expression in *Arabidopsis* are regulated by DNA methylation, predominantly found in the promoter and gene body. It is also likely that these genes are tightly epigenetically controlled until needed, maintaining high methylation frequency until the plants are infected by *R. solanacearum*. This would be similar to the reduction in methylation of defense-related genes to enhance resistance in *Arabidopsis* when it responds to *Pseudomonas syringae* PAMPs (Lee *et al*. 2023), Unfortunately, we cannot judge whether this is the case using our current data, as it was generated from uninfected plants, so future work could also focus on comparing expression and methylation profiles of these genes before and after inoculation with *R. solanacearum*.

Within the genes in common between *S. dulcamara* and the resistant *S. americanum* SP2773, we also found the uncharacterized gene Sd_g2132, which had 3 and 4 LTRs in the upstream and downstream regions respectively (compared with genome averages of 0.24 ± 0.60, *z* = 758.85 *P* < 0.001; and 0.22 ± 0.56, *z* = 1115.67 *P* < 0.001, respectively). We also saw a higher methylation frequency of this gene than the average considering all genes in the genome and all genomic locales (Supplementary Table 12; the average was 24.8 ± 32.8%, 26.9 ± 35.11%, 24.9 ± 32.9% for upstream, gene bodies, and downstream regions respectively).

It is possible that the TEs associated with the upstream region of Sd_g2132 could have a role in the transcription of this gene and to identify the role of this gene, we checked its orthologues and found it belongs to the orthogroup OG0024616, which also contains two *S. americanum* genes (sp2273chr03_g00625.1 and sp2273chr08_g01052.1) and the *S. dulcamara* gene Sd_g5379.2, which was annotated by EGGNOG as a SWI SNF-related matrix-associated actin-dependent regulator of chromatin subfamily A containing DEAD H box. Sd_g5379.2 also showed 89.14% identity (*E*-value 7E−69) to the *Capsicum annum* target of rapamycin complex (TOR) subunit LST8-1 (LOC107863575) (GenBank ID = XM_047408420.1). The TOR pathway is a central regulatory network conserved across kingdoms (Ryabova *et al*. 2019). In plants, it is involved in regulating gene expression and other metabolic adjustments controlling the switch between stress and growth, and is associated with responses to nutrients, pathogens and hormones (Ryabova *et al*. 2019). Interestingly, when the *R. solanacearum* RipA5 (former AWR5) effector was expressed in yeast, this caused effects resembling rapamycin's inhibitory effect on TOR signaling and *in planta* this effector caused a decrease in the activity of enzymes regulated by TOR (Popa *et al*. 2016). Popa *et al*. (2016) concluded that TOR-deficient plants are more susceptible to *R. solanacearum,* and that the bacterium inhibits this pathway. Our results suggest Sd_g2132 could be important during bacterial wilt infections and its transcription regulation could be associated with higher methylation and higher TE content. Altogether, these analyses identified potential epigenetic associations in genes found in common in plant species resistant to *R. solanacearum*.

### Identifying NLRs in susceptible plant species

Using comparative genomic analysis and NLR annotation, we also looked for NLR genes associated with resistant or susceptible species. Of the 239 and 434 genes classified as NLRs in S. *dulcamara* and *S. nigrum* genomes, no NLRs were found to be present in *S. dulcamara-*only orthogroups, or orthogroups shared between *S. dulcamara* and *S. americanum*. However, we did identify 53 *S. nigrum* NLRs, out of the 434 total NLRs that were shared with at least one of the other susceptible plant species (Supplementary Table 13), indicating potential susceptibility factors. We also calculated the DNA methylation frequency of these 53 genes across the genomic locales and identified one gene, Sn_g34732, which showed lower methylation in both the upstream (24.2%) and gene regions (20.9%) when compared with NLRs not shared between susceptible species, or the rest of NLRs in the *S. nigrum* genome (average of NLRs *N* = 384 was 37.0 ± 40.2%; 39.8 ± 41.6; 38.6 ± 41.5 in upstream regions, gene bodies and downstream regions respectively, Supplementary Table 14; Supplementary Fig. 4). This could suggest different epigenetic regulation of this gene compared with the rest of NLRs however, as we did not have tissue-specific RNA for *S. nigrum*, we were not able to assess the expression of this gene in this plant. Interestingly, AlphaFold results showed 96% identity with *S. americanum* Rpi-amr1b (Uniprot A0A890CMX7) encoding an NRC helper-dependent CC-NLR protein, which confers resistance to multiple strains of *Phytophthora infestans* (Witek *et al*. 2021), the causal agent of potato late blight; however, it is unknown if this NLR has a role during bacterial wilt development. Future work could look at how these 53 NLRs interact with *R. solanacearum* effectors and if their expression and DNA methylation changes after infection.

## Conclusions

We have assembled and annotated two new wild plant species genomes: *S. dulcamara* and *S. nigrum,* which showed contrasting susceptibility to the major plant pathogen *R. solanacearum*. We have assessed the methylation frequency and role of TEs across the whole genome in both species and confirmed *S. nigrum* had a higher methylation frequency than *S. dulcamara*, which is partially associated with a higher TE content and higher hypermethylation on TEs in gene bodies or upstream and downstream regions. We identified auxin-transport-related genes that are only present in *S. dulcamara*, which we hypothesize could be associated with its role as a reservoir plant for *R. solanacearum* or with resistance mechanisms. PRRs identified in common between the resistant/tolerant S*. dulcamara* and *S. nigrum* SP773, and not present in susceptible crops or wild species, and NLRs shared between susceptible plant species could also be targets for future work to validate their functional roles during disease development. Higher methylation frequency of some PRRs was also associated with lower gene expression in some plant tissues, suggesting epigenetic regulation of genes involved in disease resistance. The improved genome resources and candidate genes provided here will facilitate further investigations into the defence against this important bacterial pathogen.

## Supplementary Material

jkaf119_Supplementary_Data

## Data Availability

The genomes are available under the NCBI BioProject: PRJNA1014230 and PRJNA1017508, including raw data, and genome assemblies (JBBJLP000000000 and JAZKKB000000000). Genomes with full annotations and methylation calling are also available here: www/webfiles.york.ac.uk/Harper/Solanum_dulcamara and www/webfiles.york.ac.uk/Harper/Solanum_nigrum and in FigShare https://doi.org/10.25387/g3.29097182. Methylation profiles are also available in Gene Expression Omnibus (GEO) accession GSE262401 and GSE255584. Gene expression of the roots, stems, leaves, flowers and fruits is available in the GEO accession GSE283679. All the scripts used in this study can be found at https://github.com/sfortega/Solanaceae_genomes. Supplemental material available at G3 online.
