# Supplementary material for: Assembly and annotation of Solanum dulcamara and Solanum nigrum plant genomes, two nightshades with contrasting susceptibilities to Ralstonia solanacearum
Source: G3 (Bethesda). 2025 May 26;15(7):jkaf119. doi: 10.1093/g3journal/jkaf119 (PMC12239606; doi:10.1093/g3journal/jkaf119)
Supplement: jkaf119_Supplementary_Data [file jkaf119_supplementary_data.zip › Supplemental_Material_Legends_G3-2025-405851.docx]

**Supplementary figures legends**

**Figure S1. Pseudochromosome assembly using the *S. dulcamara* genome by** (Christenhusz 2023) **as reference (**“S.Dulc1”)**.** **A**. Twelve pseudochromosome scaffolds (“Sd_”) using 3169 contigs of the new *S. dulcamara* genome, obtained by ntJoin using the options k=16 and w=500, n=2, followed by mapping between both assemblies with nucmer with the options -c 500 -b 100 -l 1000. B. Twelve pseudochromosome scaffolds (“Sn_”) using 654 contigs of the *S. nigrum* genome obtained by ntJoin using the options k=32 and w=200, n=2, followed by mapping between both assemblies with nucmer with the options -c 32 -b 100 -l 100 and filtered to maintain only high identity mapping (≥93% and with a minimum length of 1000 bp).

**Figure S2. Pearson correlation between TE content in different genomic locales of the *S. dulcamara* and *S. nigrum* genes.** A - C. Pearson correlation and p-value of upstream (1 kbp; A), gene body (B) and downstream (1 kbp; C) regions containing TIRs. D - F. Pearson correlation and p-value of upstream (1 kbp; D), gene body (E) and downstream (1 kbp; F) regions containing non-TIRs. G - I. Pearson correlation and p-value of upstream (1 kbp; G), gene body (H) and downstream (1 kbp; I) regions containing LTRs. J - K. Pearson correlation and p-value of upstream (1 kbp; J), gene body (K) and downstream (1 kbp; L) regions in genes without TEs.

**Figure S3. Gene expression (log (TPMs+1)) of Sd_g8870 and Sd_g28574**. The expression as measured in roots (grey), stem (green), leaves (green), flowers (purple) and berries (red) of selected PRRs compared with the rest of leucine-rich repeat receptor-like kinase (LRRs).

**Figure S4. DNA methylation frequency of *S. nigrum* NLRs across gene body and upstream and downstream regions,** comparing NLRs shared with susceptible plant species (52 NLR in green and Sd_g34732 in blue) and the rest of NLRs (N=384 in grey). Sd_g34732 showed significantly lower DNA methylation frequency than the average of *S. nigrum* NLRs, suggesting distinct epigenetic regulation.

**Supplementary tables**

**Table S1.** Seqkit stats of the S. dulcamara and S. nigrum genomes.

**Table S2**. *S. dulcamara* and *S. nigrum* pseudochromosome sizes and the number of contigs in each using S. dulcamara genome by (Christenhusz 2023) as reference.

**Table S3.** Methylation frequencies in each contig of the *S. dulcamara* genome.

**Table S4.** Methylation frequencies in each contig of the *S. nigrum* genome.

**Table S5.** Orthogroups are assigned using SYNIMA tool. Gene names correspond with the different annotations of the genomes: "C88" corresponds with the potato genome (*S. tuberosum* C88; http://spuddb.uga.edu/data/), "Solyc" corresponds with the tomato genome (pangenome of *S. lycopersicum*; Zhou et al., 2022), "SMEL4.1" corresponds with the aubergine genome (*S. melongena*; https://solgenomics.net/ftp/genomes/Solanum_melongena_V4.1), "sp2273" corresponds wiht the resistant *S. americanum* SP2773 accession and "SaSP2275" with the susceptible *S. americanum* accession (Moon et al., 2021), "Sd" corresponds with the *S. dulcamara* annotations and "Sn" with the *S. nigrum* genome.

**Table S6.** OrthoFinder statistics of the orthogroups of the analysis with 7 plant species (resistant/tolerant species: *S. dulcamara* and *S. americanum* SP2773 and susceptible species: *S. nigrum*, *S. americanum* SP2775, *S. lycopersicum*, *S. melongena* and *S. tuberosum*) .

**Table S7.** Number of common orthogroups between with 7 plant species (resistant/tolerant species: *S. dulcamara* and *S. americanum* SP2773 and susceptible species: *S. nigrum*, *S. americanum* SP2775, *S. lycopersicum*, *S. melongena* and *S. tuberosum*).

**Table S8.** Orthofinder statistics of the orthogroups and genes in each specie (resistant/tolerant species: *S. dulcamara* and *S. americanum* SP2773 and susceptible species: *S. nigrum*, *S. americanum* SP2775, *S. lycopersicum*, *S. melongena* and *S. tuberosum*).

**Table S9**. Genes in 27 orthogroups only represented by genes from *S. dulcamara.*

**Table S10**. Gene ontology enrichment of 90 genes belonging to 27 orthogroups represented only by *S. dulcamara* genes, and the genes in orthogroups in common between *S. dulcamara* and *S. americanum* SP2773, both resistant to *R. solanacearum*.

**Table S11**. Genes in 19 orthogroups in common between *S. dulcamara* and *S. americanum* SP2773.

**Table S12**. Methylation frequency across gene body, 1Kbp upstream and 1Kbp downstream for the genes in common between resistant plant species to *R. solanacearum: S. dulcamara* *and* *S. americanum SP2773*. In red, significantly higher methylation frequency than the average methylation frequency in other leucine-repeat proteins (LRRs). In blue, significantly lower methylation frequency than the average methylation frequency in other LRRs.

**Table S13.** Orthogroups with NLRs shared between susceptible plants species. Gene names correspond with the different annotations of the genomes: "C88" corresponds with the potato genome (S. tuberosum C88; http://spuddb.uga.edu/data/), "Solyc" corresponds with the tomato genome (pangenome of S. lycopersicum; Zhou et al., 2022), "SMEL4.1" corresponds with the aubergine genome (S. melongena; https://solgenomics.net/ftp/genomes/Solanum_melongena_V4.1), "sp2273" corresponds wiht the resistant S. americanum SP2773 accession and "SaSP2275" with the susceptible S. americanum accession (Moon et al., 2021), "Sd" corresponds with the S. dulcamara annotations and "Sn" with the S. nigrum genome.

**Table S14**. Methylation frequency across gene body, 1Kbp upstream and 1Kbp downstream for the *S. nigrum* NLRs genes in common between susceptible plant species to *R. solanacearum: S. nigrum, S. americanum* SP2775, *S. lycopersicum, S. melongena* and *S. tuberosum*. In blue, significantly lower methylation frequency than the average methylation frequency in other NLRs.
