# Supplementary figures and images for: Assembly and annotation of Solanum dulcamara and Solanum nigrum plant genomes, two nightshades with contrasting susceptibilities to Ralstonia solanacearum
Source: G3 (Bethesda). 2025 May 26;15(7):jkaf119. doi: 10.1093/g3journal/jkaf119 (PMC12239606; doi:10.1093/g3journal/jkaf119)

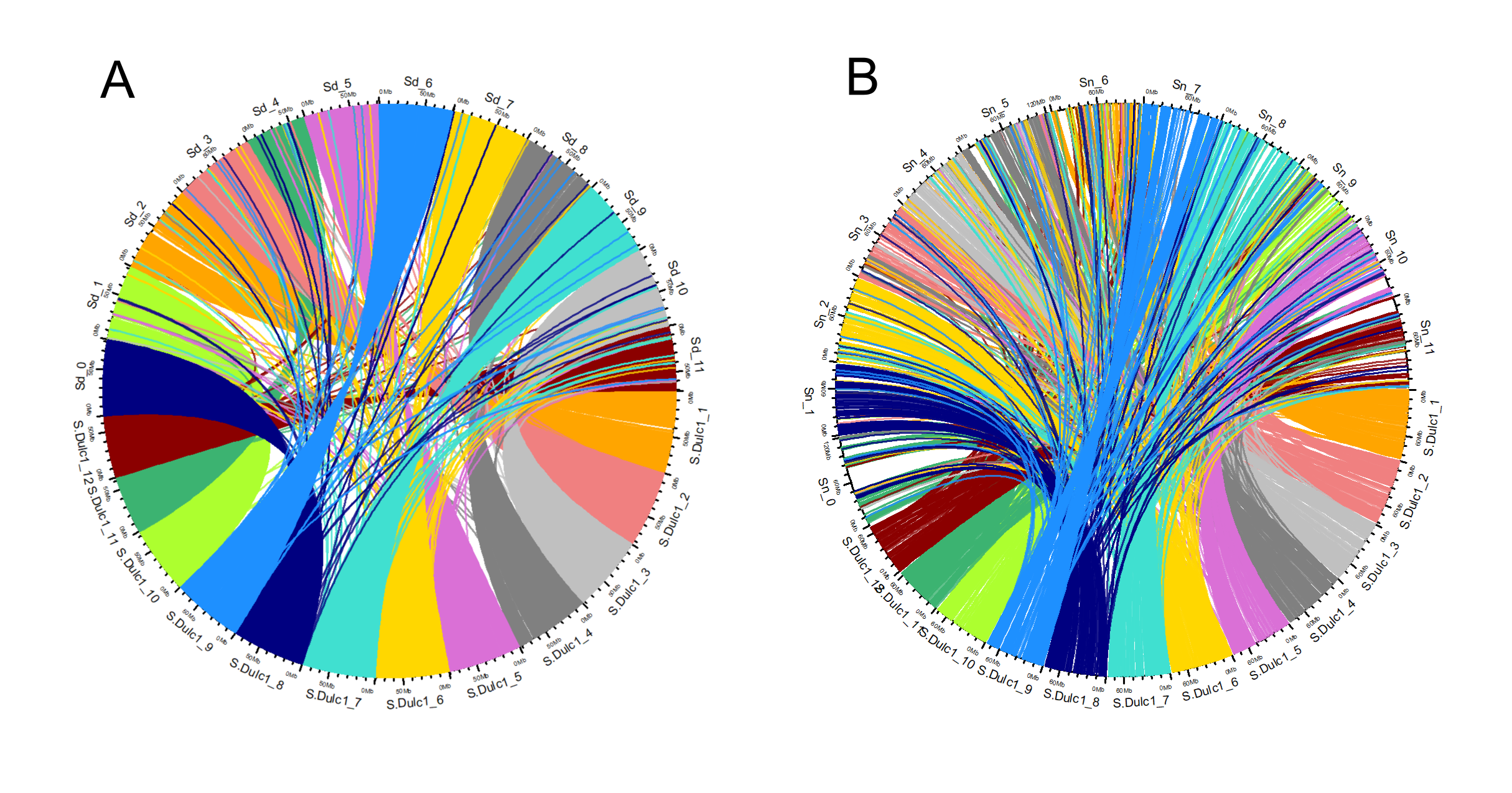

Supplement: jkaf119_Supplementary_Data [file jkaf119_supplementary_data.zip › Figure_S1_G3-2025-405851.png]

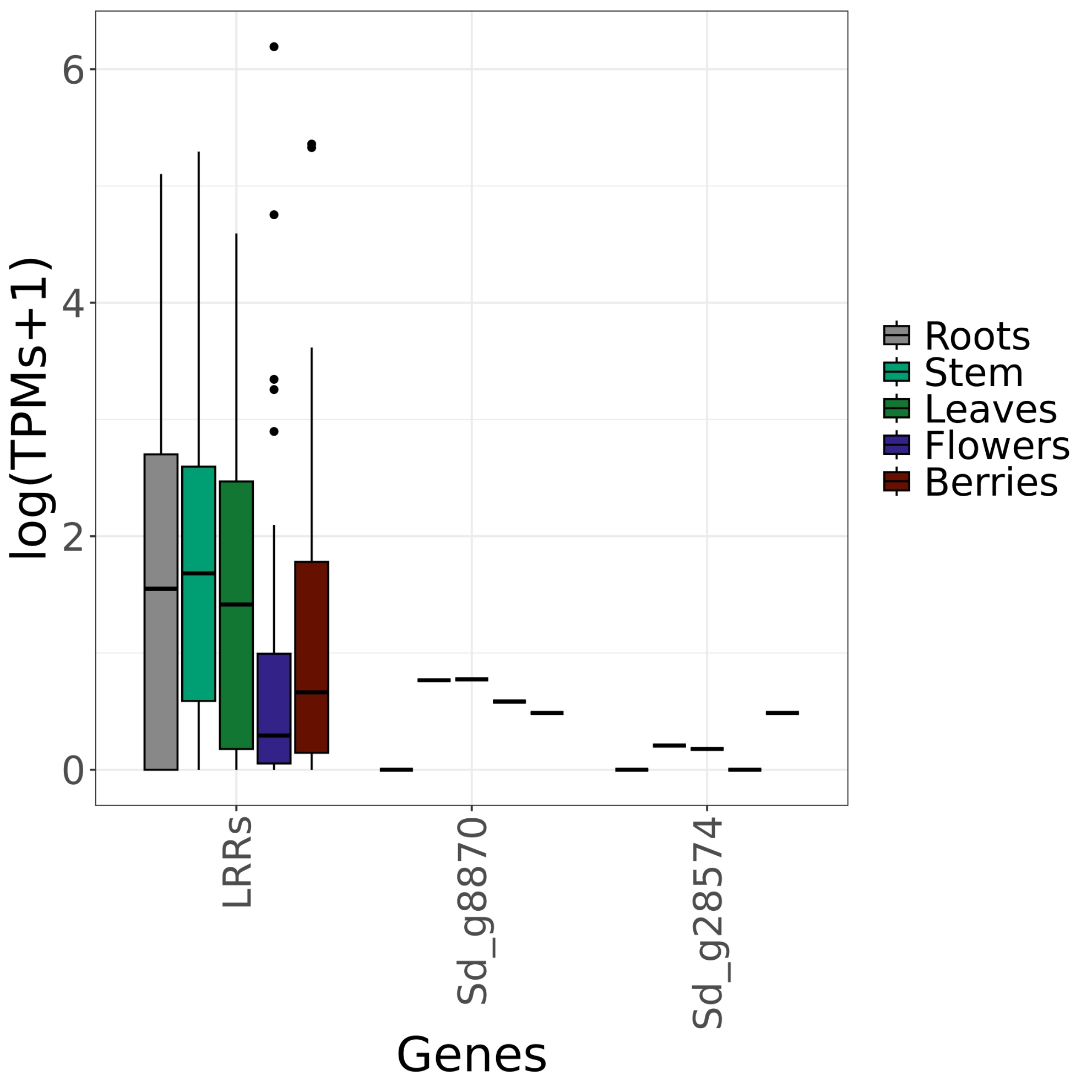

Supplement: jkaf119_Supplementary_Data [file jkaf119_supplementary_data.zip › Figure_S3_G3-2025-405851.png]

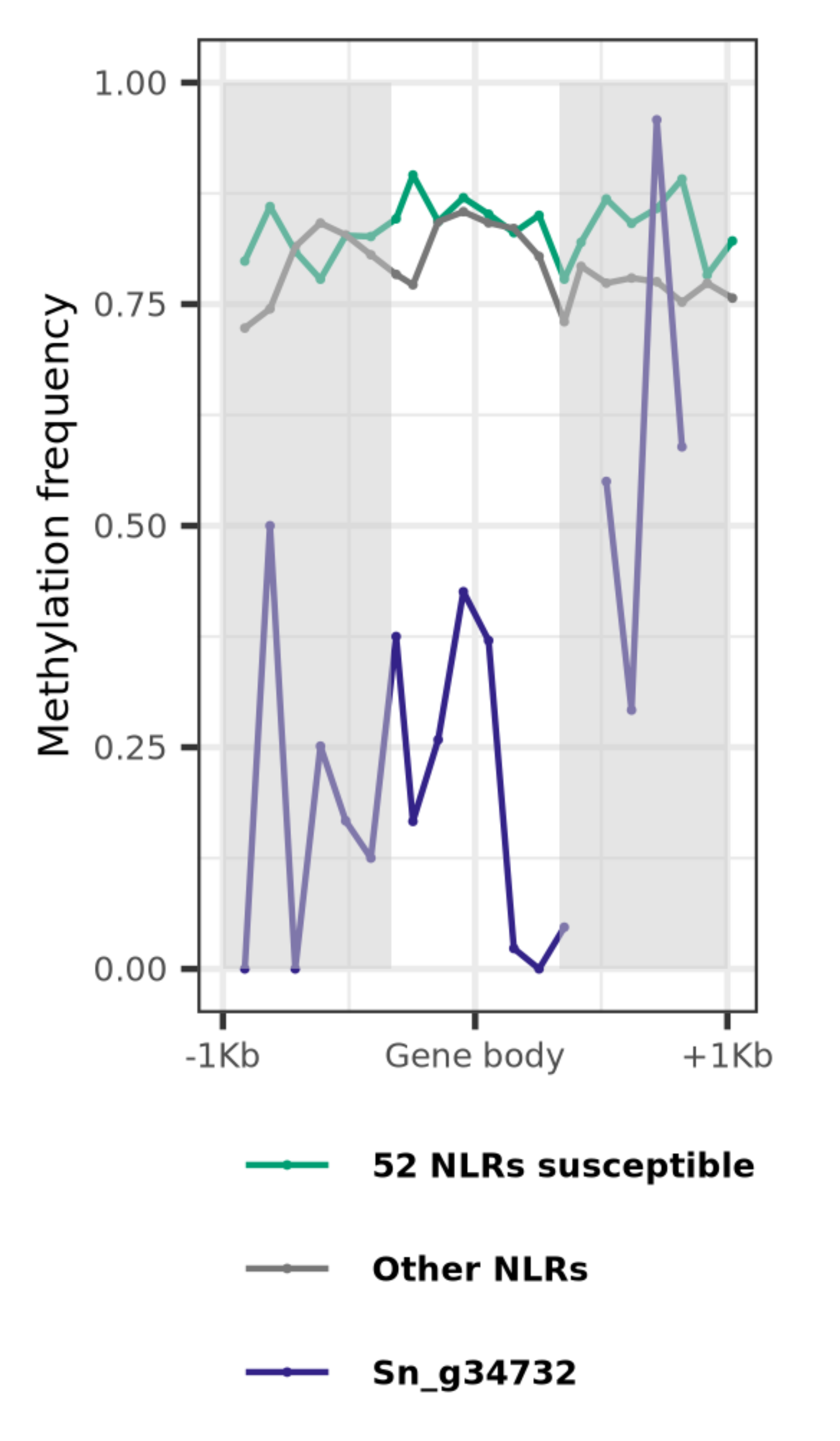

Supplement: jkaf119_Supplementary_Data [file jkaf119_supplementary_data.zip › Figure_S4_G3-2025-405851.png]
